# Parental Knowledge, Attitudes, and Practices Regarding Early Childhood Caries in Bihor, Romania: A Cross-Sectional Study

**DOI:** 10.3390/children11091131

**Published:** 2024-09-18

**Authors:** Abel Emanuel Moca, Raluca Iulia Juncar, Rahela Tabita Moca, Mihai Juncar, Rebeca Daniela Marton, Luminița Ligia Vaida

**Affiliations:** 1 Department of Dentistry, Faculty of Medicine and Pharmacy, University of Oradea, 10 Piața 1 Decembrie Street, 410073 Oradea, Romania; abelmoca@yahoo.com (A.E.M.); mihaijuncar@gmail.com (M.J.); ligia_vaida@yahoo.com (L.L.V.); 2 Doctoral School of Biomedical Sciences, University of Oradea, 1 Universității Street, 410087 Oradea, Romania; rebeca.marton@yahoo.com

**Keywords:** parental knowledge, attitudes and practices, early childhood caries, caries prevention

## Abstract

Background/Objectives: Early childhood caries (ECC) is a significant global health issue, particularly affecting deciduous teeth in young children. ECC is prevalent in Romania, where nearly half of children experience dental caries. This study aimed to assess the influence of gender, age, and living environment on parents’ knowledge, attitudes, and practices (KAP) regarding ECC in Bihor County, Romania, using a KAP-type questionnaire. Methods: A cross-sectional study was conducted between March and September 2024, utilizing an online KAP questionnaire distributed via social networks. The sample comprised 419 parents of children under six years old. Respondents provided socio-demographic data and answered questions regarding their knowledge, attitudes, and practices concerning ECC prevention, treatment, and oral health. Statistical analysis was conducted using Chi-square tests to assess associations between demographic factors and parental KAP. A *p*-value of less than 0.05 was considered statistically significant. Results: Of the 419 respondents, 83.1% were female, and 62.5% were between the ages of 31 and 40. Significant gender-based differences were found in knowledge about pain from caries (93.7% of female participants vs. 81.7% of male participants, *p* = 0.004) and the importance of brushing twice daily (93.7% of female respondents vs. 80.3% of male respondents, *p* = 0.010). Younger parents (aged 18–30) were more likely to affirm the effectiveness of fluoride (65.4%) compared to those aged 31–40 (53.1%, *p* = 0.02). Urban parents were more likely to correctly identify the timing of tooth eruption (59.1% vs. 52.6% of rural parents, *p* = 0.021). Conclusions: The study highlights gender, age, and urban–rural disparities in parental knowledge and attitudes towards ECC. These findings underscore the need for targeted educational interventions to improve oral health outcomes and reduce ECC prevalence in the Bihor region. Tailored public health strategies addressing demographic factors could enhance preventive oral health behaviors and reduce the healthcare burden associated with untreated dental caries.

## 1. Introduction

Dental caries is a biofilm-mediated disease, exacerbated by carbohydrate intake, multifactorial in nature, and dynamic in its progression, resulting in alternating phases of demineralization and remineralization of hard dental tissues [1]. This complex condition is influenced by a variety of factors, including socioeconomic, demographic, behavioral, and biological determinants [2]. Despite sustained efforts to reduce the prevalence of dental caries, it remains a pervasive disease globally [3]. Currently, more than one-third of the world’s population lives with untreated dental caries, with a prevalence of 29% in permanent teeth and 43% in deciduous teeth [4]. The consequences of untreated dental caries are multidimensional, affecting not only overall health [5] but also the patient’s quality of life, with significant social, psychological, and biological implications [6]. The high prevalence, negative impact on patient well-being, and associated treatment costs make dental caries a significant global health burden [7].

The significant prevalence of dental caries among the pediatric population, particularly in deciduous teeth, has prompted increased attention to this demographic [8]. Early childhood caries (ECC) is defined as the presence of one or more decayed (non-cavitated or cavitated lesions), missing, or filled (due to caries) surfaces in any primary tooth of a child under six years of age [9]. Several specific factors contribute to the risk of ECC, including the frequency, amount, and timing of sugar consumption, prolonged breastfeeding, parental leniency regarding the child’s tooth brushing, and the lack of evening tooth brushing [10]. Due to the early age at which ECC develops, the initial stages are often overlooked by caregivers, leading to dental visits only when the caries have progressed to the pulp chamber of the primary tooth, causing pain during mastication, spontaneous nocturnal pain, or persistent discomfort [11]. Given the immediate and long-term consequences of ECC, and considering the early age of the patients, treatment can be complex and difficult to perform chairside, often necessitating general anesthesia for effective management [12].

Therefore, emphasis must be placed on the prevention of ECC [11], alongside educating parents and children on appropriate oral hygiene practices, proper nutrition, and awareness of ECC risk factors [13]. To design effective educational programs targeting caregivers and parents, it is essential to assess their educational needs.

A knowledge, attitude, and practice (KAP) survey is a valuable instrument for assessing the level of knowledge within a specific health domain. Originally developed in the 1950s in the fields of family planning and population research, KAP surveys have since gained widespread acceptance as an effective tool for investigating health-related behaviors and practices. These surveys are straightforward to design, administer, analyze, and interpret [14]. The KAP questionnaire serves as a representative study of a general population, aiming to assess what individuals know (knowledge), believe (attitudes), and do (practices) concerning a particular subject. This tool facilitates the collection of both qualitative and quantitative data [14]. Its use in studying ECC has become increasingly common across various global populations [15,16,17], contributing to the development of an international database that provides a comprehensive perspective on parental knowledge, attitudes, and practices related to ECC.

In Romania, the prevalence of dental caries in deciduous teeth surpasses the global average reported by the World Health Organization (WHO), reaching 48.2% [4]. Furthermore, the use of emergency dental services is generally high among the pediatric population in Romania [18]. However, there is a notable lack of studies investigating the knowledge, attitudes, and practices of Romanian parents concerning deciduous teeth and ECC. At the time of this study’s conception, no similar research had been identified within the Romanian population. Understanding how Romanian parents perceive and manage their children’s oral health is crucial for identifying specific areas where education and intervention are needed. This research is essential for developing targeted public health initiatives that can reduce the incidence of ECC, improve children’s overall health outcomes, and ultimately decrease the long-term healthcare burden associated with untreated dental caries.

We hypothesize that demographic factors such as gender, age, and living environment will influence, at least in some measure, the KAP of parents in Bihor, Romania, regarding ECC. Therefore, this study aims to investigate the influence of gender, age, and living environment on the KAP of parents in Bihor, Romania, concerning various aspects of ECC, including prevention, dental visits, treatment of caries in deciduous teeth, and proper diet. By utilizing a KAP-type questionnaire, the research seeks to assess how these demographic factors shape parents’ understanding and management of ECC. The findings will contribute to the development of targeted public health initiatives aimed at reducing the incidence of ECC and improving oral health outcomes in Bihor, Romania.

## 2. Materials and Methods

### 2.1. Ethical Considerations

This research was conducted in compliance with the ethical standards outlined in the 1964 Declaration of Helsinki and its subsequent amendments. The study received approval from the Research Ethics Committee of the University of Oradea (No. CEFMF/02 from 30 August 2024). Prior to completing the questionnaire, respondents were informed that participation was entirely voluntary and anonymous, with no financial or other incentives provided. Consent was implied through the voluntary completion of the questionnaire.

### 2.2. Participants and Data Collection

The study was conducted between 1 March 2024 and 1 September 2024 as a cross-sectional study over a six-month period. A knowledge, attitude, and practice (KAP) questionnaire was administered using the online platform www.survio.com (accessed on 1 March 2024) (Survio s.r.o., Brno, Czech Republic), a tool designed for online surveys. The questionnaire link was distributed via social networks to maximize the reach within the target region. The KAP questionnaire on ECC was based on the framework proposed by Mani et al. (2012) [19], with modifications to suit the study context.

The KAP questionnaire used in this study was translated and adapted in Romanian and was designed to assess the knowledge, attitudes, and practices of parents regarding ECC. The questionnaire was divided into four sections, as outlined below:

- Section one—Demographic Information: The first section gathered socio-demographic details, including gender, age, ethnicity, marital status, living environment (urban or rural), education level, and number of children (Table 1). These data were analyzed to explore potential associations with parents’ knowledge, attitudes, and practices regarding ECC.
- Section two—Knowledge Section (items 1–15): This section aimed to evaluate parents’ understanding of key ECC-related topics such as tooth eruption, the role of fluoride, diet, and dental visits. Participants responded with “Yes”, “No”, or “Not sure”. In the knowledge section of the questionnaire, each correct answer was assigned a score of 1, while incorrect or ‘Not sure’ responses were scored as 0. Participants’ overall knowledge was classified into three levels based on the total number of correct answers: low (0–5 correct answers), moderate (6–10 correct answers), and high (11–15 correct answers). All questions from the knowledge section are presented in Table 2 in the Section 3.
- Section three—Attitude Section (items 16–22): The attitude section focused on understanding parents’ beliefs regarding ECC prevention, brushing habits, and the importance of dental visits. A 5-point Likert scale ranging from “Strongly disagree” to “Strongly agree” was used, with “Not sure” as an additional option. This section helped assess the strength of parental commitment to maintaining their child’s oral health. Attitude-related questions appear in Table 3 of the Section 3.
- Section four—Practice Section (items 23–32): This section examined how frequently parents engaged in oral hygiene practices and dietary management for their children, as well as how often they visited the dentist. Participants answered on a 5-point scale: “Never”, “Sometimes”, “Often”, “Always”, and “Not sure”. Higher scores indicated more frequent engagement in preventive oral health behaviors. Practice-related questions can be found in Table 4 in the Section 3.

Participants were required to meet the following inclusion criteria: being a parent or caregiver of at least one child under the age of six, being a Romanian speaker, and residing in Bihor County at the time of completing the questionnaire.

Participants were excluded if they were not the parent or primary caregiver of a child under six, were non-Romanian speakers, did not reside in Bihor County at the time of data collection, had incomplete responses, or had formal dental training.

### 2.3. Sample Size Calculation

The sample size calculation was performed using Python 3 (Python Software Foundation, Wilmington, DE, USA). To determine the appropriate sample size for the study, a sample size calculation was performed. We aimed for a 95% confidence level, corresponding to a Z-score of approximately 1.96, and a margin of error of 5%, a standard in scientific research to balance precision and feasibility. Assuming a population proportion of 0.5, which provides the maximum required sample size, the initial sample size was calculated using the following formula:

n = Z^2^⋅p⋅(1 − p)/E^2^,

where Z is the Z-score, p is the population proportion, and E is the margin of error.

Considering the finite population size, the sample size was adjusted using the finite population correction equation shown below:

n_adjusted_ = n/1 + (n − 1/N),

where N is the population size [20]. The final adjusted sample size was calculated to be approximately 377. This sample size ensures the representativeness of the results for the entire population with the specified confidence level and margin of error.

### 2.4. Statistical Analysis

The statistical analysis for this study was conducted using Python (Python Software Foundation, Wilmington, DE, USA). Data were recorded and organized in Microsoft Excel 2013 (Microsoft, Redmond, WA, USA), which was also used for basic data handling, such as sorting and calculating summary statistics (e.g., means, percentages). Microsoft Word 2013 (Microsoft, Redmond, WA, USA) was used for drafting, formatting, and presenting the final report of the study.

The Chi-square test of independence was used to assess associations between categorical variables. This test was selected due to the categorical nature of the data, analyzing two variables at a time to determine if there is a significant association between them. A significant result (*p* < 0.05) indicates that the distribution of responses for an item is significantly different between groups, while a non-significant result suggests no strong evidence of a difference between groups.

Following a significant result from the Chi-square test, a post hoc analysis was conducted by calculating the adjusted residuals for each cell in the contingency table. Residuals with an absolute value greater than 1.96 were considered significant, indicating that certain gender-response combinations contributed disproportionately to the overall Chi-square result. This method enabled the identification of specific patterns of association within the data.

## 3. Results

### 3.1. Socio-Demographic Characteristics

The online questionnaire was accessed by 798 respondents, of whom 419 completed it, yielding a completion rate of 52.5%. The majority of respondents were female (83.1%, *n* = 348) and aged between 31 and 40 years (62.5%, *n* = 262). Most participants identified as Romanian (92.8%, *n* = 389) and resided in urban areas (67.8%, *n* = 284). Table 1 provides a detailed overview of the demographic characteristics of the respondent group.

**Table 1:** Respondents’ distribution according to different variables.

Variable	Size	
	No.	Percentage
**Gender**		
Male	71	16.9%
Female	348	83.1%
**Age**		
18–30 y	104	24.8%
31–40 y	262	62.5%
>40 y	53	12.7%
**Ethnicity**		
Romanian	389	92.8%
Hungarian	25	6.0%
Roma	3	0.7%
Slovak	2	0.5%
**Marital status**		
Not married	18	4.3%
Married	385	91.9%
Divorced	13	3.1%
Widowed	3	0.7%
**Living environment**		
Urban	284	67.8%
Rural	135	32.2%
**Formal education**		
Middle school	1	0.3%
High school	95	22.4%
Bachelor’s degree	192	45.9%
Master’s degree	119	28.5%
PhD	12	2.9%
**Number of children**		
1	190	45.4%
2	160	38.2%
3	45	10.7%
4 or more	24	5.7%

### 3.2. Gender, Age, Living Environment, and Parental Knowledge Regarding ECC

**Figure 1:**
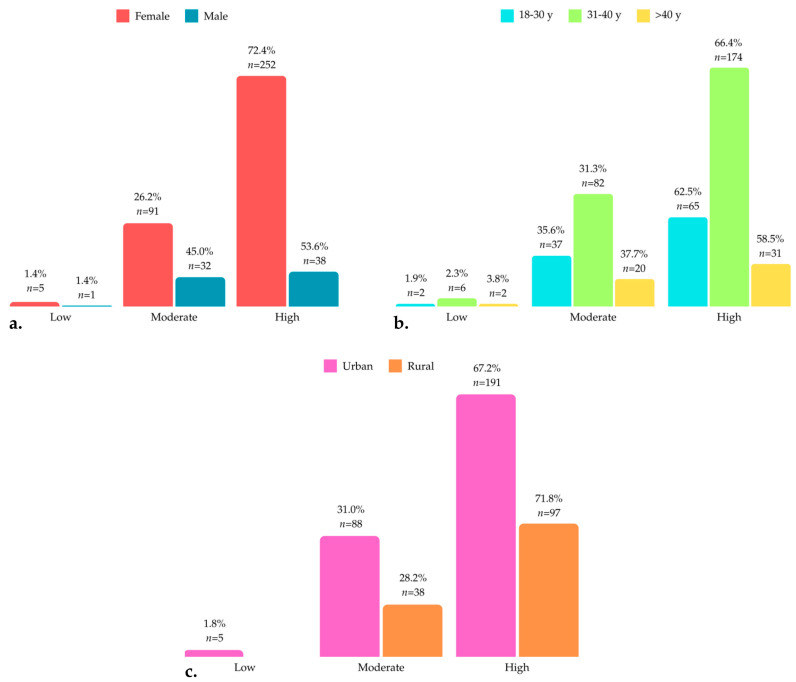
The respondents’ overall knowledge according to gender (**a**), age (**b**), and living environment (**c**).

In the knowledge section, most participants demonstrated high levels of knowledge, although differences were observed across gender, age, and living environment. Among females, 72.4% had high knowledge, while 26.2% scored moderately, and only 1.4% had low knowledge. For males, 53.6% had high knowledge, 45.0% moderate, and 1.4% low. Age-wise, the 31–40 group had the highest proportion of high knowledge (66.4%), with 31.3% scoring moderate and 2.3% low. Similarly, participants aged 18–30 and over 40 showed significant moderate knowledge (35.6% and 37.7%, respectively). Urban participants displayed 67.2% high, 31.0% moderate, and 1.8% low knowledge, while rural respondents showed slightly more high knowledge (71.8%) and 28.2% moderate, with no low scores recorded (Figure 1).

Table 2 presents the distribution of respondents based on items assessing parental knowledge regarding the prevention of ECC, as well as other related information. Statistically significant gender-based differences were observed in responses to items 4 (*p* = 0.004), 7 (*p* = 0.010), 8 (*p* = 0.000), and 11 (*p* = 0.029), as follows:

- Item 4—“Children may experience pain due to cavities in their primary teeth”: Gender significantly influenced the respondents’ certainty or agreement with the understanding that children can experience pain from decay in their deciduous teeth. A higher percentage of female respondents (93.7%) responded “Yes”, indicating certainty, compared to male respondents (81.7%). Conversely, male respondents exhibited greater uncertainty, with 15.5% responding “Not sure”, compared to 5.4% of female respondents.
- Item 7—“Primary teeth should be brushed from the outset with fluoride toothpaste”: Gender influenced certainty or agreement with the practice of brushing deciduous teeth with fluoride toothpaste from their eruption. Female respondents demonstrated higher certainty (57.76% responded “Yes”) compared to male respondents (50.70% responded “Yes”). Male participants showed notably more uncertainty (22.54% responded “Not sure”) than female participants (9.77%).
- Item 8—“Brushing should be performed twice daily from the beginning”: Gender also influenced certainty or agreement with the recommendation to brush teeth twice daily from the eruption of the first tooth. Female respondents demonstrated higher certainty (93.7% answered “Yes”) compared to male respondents (80.3%). Male respondents again showed greater uncertainty (12.6% responded “Not sure”) compared to female respondents (2.6%).
- Item 11—“Breastfeeding can contribute to the development of dental caries”: Gender influenced certainty regarding the statement that breastfeeding can cause tooth decay. A greater proportion of female respondents disagreed with this statement (50.6% responded “No”) compared to male respondents (39.4%). Male respondents displayed higher levels of uncertainty, with 38.1% responding “Not sure”, in contrast to 23.0% of female respondents.

**Table 2:** Distribution of respondents by gender, age, and living environment, and their responses to knowledge (K) items.

Answer	Gender		Age			Living Environment	
	Male	Female	18–30 y	31–40 y	>40 y	Urban	Rural
**Item 1: Primary teeth typically begin to erupt around the age of 6 months, though they may emerge earlier.**							
Yes	59 (83.1%)	305 (87.6%)	47 (88.6%)	182 (87.9%)	31 (81.6%)	244 (85.9%)	120 (88.9%)
No	7 (9.9%)	27 (7.8%)	3 (5.7%)	15 (7.3%)	4 (10.5%)	24 (8.5%)	9 (6.7%)
Not sure	5 (7.0%)	16 (4.6%)	3 (5.7%)	10 (4.8%)	3 (7.9%)	16 (5.6%)	6 (4.4%)
*p*	0.559		0.833			0.701	
**Item 2: The final permanent tooth typically erupts between the ages of 11 and 12 years.**							
Yes	35 (49.3%)	204 (58.6%)	64 (61.5%)	150 (57.3%)	25 (47.1%)	168 (59.1%)	71 (52.6%)
No	7 (9.9%)	37 (10.6%)	8 (7.7%)	26 (9.9%)	10 (18.9%)	35 (12.3%)	9 (6.7%)
Not sure	29 (40.8%)	107 (30.8%)	32 (30.8%)	86 (32.8%)	18 (34.0%)	81 (28.6%)	55 (40.7%)
*p*	0.248		0.214			0.021	
**Item 3: Dental caries can affect primary teeth immediately after they erupt.**							
Yes	55 (77.5%)	300 (86.2%)	70 (67.3%)	180 (68.7%)	35 (66.0%)	236 (83.1%)	119 (88.1%)
No	4 (5.6%)	18 (5.2%)	10 (9.6%)	30 (11.5%)	8 (15.1%)	19 (6.7%)	3 (2.3%)
Not sure	12 (16.9%)	30 (8.6%)	24 (23.1%)	52 (19.8%)	10 (18.9%)	29 (10.2%)	13 (9.6%)
*p*	0.101		0.501			0.151	
**Item 4: Children may experience pain due to cavities in their primary teeth.**							
Yes	326 (93.7%)	58 (81.7%)	80 (77.0%)	200 (76.3%)	40 (75.5%)	270 (95.0%)	130 (96.3%)
No	3 (0.9%)	2 (2.8%)	12 (11.5%)	32 (12.2%)	6 (11.3%)	5 (1.8%)	2 (1.5%)
Not sure	19 (5.4%)	11 (15.5%)	12 (11.5%)	30 (11.5%)	7 (13.2%)	9 (3.2%)	3 (2.2%)
*p*	0.004		0.463			0.058	
**Item 5: Untreated dental caries can lead to abscesses.**							
Yes	66 (93.0%)	321 (92.2%)	90 (86.5%)	220 (83.9%)	45 (84.9%)	230 (81.0%)	110 (81.5%)
No	0 (0.0%)	1 (0.3%)	6 (5.8%)	20 (7.6%)	5 (9.4%)	30 (10.6%)	15 (11.1%)
Not sure	5 (7.0%)	26 (7.5%)	8 (7.7%)	22 (8.5%)	3 (5.7%)	24 (8.4%)	10 (7.4%)
*p*	0.895		0.111			0.248	
**Item 6: Oral hygiene should be maintained even before the eruption of teeth.**							
Yes	50 (70.4%)	279 (80.2%)	100 (96.2%)	240 (91.6%)	50 (94.3%)	223 (78.5%)	37 (56.0%)
No	10 (14.1%)	26 (7.5%)	2 (1.9%)	12 (4.6%)	2 (3.8%)	25 (8.8%)	11 (16.7%)
Not sure	11 (15.5%)	43 (12.3%)	2 (1.9%)	10 (3.8%)	1 (1.9%)	36 (12.7%)	18 (27.3%)
*p*	0.125		0.669			0.963	
**Item 7: Primary teeth should be brushed from the outset with fluoride toothpaste.**							
Yes	201 (57.8%)	36 (50.7%)	58 (55.8%)	154 (58.8%)	25 (47.2%)	210 (74.0%)	100 (74.1%)
No	113 (32.5%)	19 (26.8%)	31 (29.8%)	80 (30.5%)	21 (39.6%)	40 (14.0%)	20 (14.8%)
Not sure	34 (9.7%)	16 (22.5%)	15 (14.4%)	28 (10.7%)	7 (13.2%)	34 (12.0%)	15 (11.1%)
*p*	0.010		0.509			0.066	
**Item 8: Brushing should be performed twice daily from the beginning.**							
Yes	326 (93.7%)	57 (80.3%)	93 (89.4%)	243 (92.7%)	47 (88.6%)	270 (95.0%)	130 (96.3%)
No	13 (3.7%)	5 (7.1%)	6 (5.8%)	8 (3.1%)	3 (5.7%)	5 (1.8%)	2 (1.5%)
Not sure	9 (2.6%)	9 (12.6%)	5 (4.8%)	11 (4.2%)	3 (5.7%)	9 (3.2%)	3 (2.2%)
*p*	0.000		0.556			0.315	
**Item 9: Parents should brush their child’s teeth, even if the child resists.**							
Yes	65 (91.5%)	320 (92.0%)	93 (89.4%)	243 (92.7%)	47 (88.6%)	263 (92.6%)	122 (90.4%)
No	5 (7.1%)	21 (6.0%)	6 (5.8%)	8 (3.1%)	3 (5.7%)	16 (5.6%)	10 (7.4%)
Not sure	1 (1.4%)	7 (2.0%)	5 (4.8%)	11 (4.2%)	3 (5.7%)	5 (1.8%)	3 (2.2%)
*p*	0.900		0.410			0.735	
**Item 10: The administration of fluoride is an effective method for preventing cavities.**							
Yes	36 (50.7%)	204 (58.6%)	68 (65.4%)	139 (53.1%)	33 (62.3%)	230 (81.0%)	110 (81.5%)
No	11 (15.5%)	35 (10.1%)	9 (8.7%)	27 (10.3%)	10 (18.9%)	30 (10.6%)	15 (11.1%)
Not sure	24 (33.8%)	109 (31.3%)	27 (25.9%)	96 (36.6%)	10 (18.8%)	24 (8.4%)	10 (7.4%)
*p*	0.309		0.020			0.076	
**Item 11: Breastfeeding can contribute to the development of dental caries.**							
Yes	92 (26.4%)	16 (22.5%)	24 (23.1%)	68 (26.0%)	16 (30.2%)	72 (25.4%)	36 (26.7%)
No	176 (50.6%)	28 (39.4%)	56 (53.8%)	140 (53.4%)	30 (56.6%)	141 (49.6%)	63 (46.6%)
Not sure	80 (23.0%)	27 (38.1%)	24 (23.1%)	54 (20.6%)	7 (13.2%)	71 (25.0%)	36 (26.7%)
*p*	0.029		0.856			0.849	
**Item 12: The consumption of sweets can lead to dental caries.**							
Yes	71 (100.0%)	342 (98.2%)	102 (98.0%)	258 (98.4%)	53 (100.0%)	270 (95.0%)	130 (96.3%)
No	0 (0.0%)	3 (0.9%)	1 (1.0%)	2 (0.8%)	0 (0.0%)	5 (1.8%)	2 (1.5%)
Not sure	0 (0.0%)	3 (0.9%)	1 (1.0%)	2 (0.8%)	0 (0.0%)	9 (3.2%)	3 (2.2%)
*p*	0.537		0.507			0.998	
**Item 13: Drinking sweetened beverages from a bottle can cause dental caries.**							
Yes	61 (85.9%)	317 (91.1%)	96 (92.3%)	240 (91.6%)	42 (79.2%)	213 (75.0%)	113 (83.7%)
No	1 (1.4%)	9 (2.6%)	3 (2.9%)	6 (2.3%)	3 (5.7%)	4 (12.0%)	8 (5.9%)
Not sure	9 (12.7%)	22 (6.3%)	5 (4.8%)	16 (6.1%)	8 (15.1%)	37 (13.0%)	14 (10.4%)
*p*	0.154		0.684			0.206	
**Item 14: The first visit to the dentist should occur around the age of 1 year.**							
Yes	36 (50.7%)	206 (59.2%)	64 (61.5%)	153 (58.4%)	25 (47.2%)	168 (59.2%)	74 (54.8%)
No	13 (18.3%)	38 (10.9%)	9 (8.7%)	32 (12.2%)	10 (18.8%)	38 (13.4%)	13 (9.6%)
Not sure	22 (31.0%)	104 (29.9%)	31 (29.8%)	77 (29.4%)	18 (34.0%)	78 (27.4%)	48 (35.6%)
*p*	0.182		0.328			0.188	
**Item 15: Cavities in primary teeth require immediate treatment upon detection.**							
Yes	52 (73.2%)	274 (78.7%)	74 (71.2%)	210 (80.2%)	42 (79.2%)	213 (75.0%)	113 (83.7%)
No	9 (12.7%)	33 (9.5%)	12 (11.5%)	24 (9.2%)	6 (11.3%)	34 (12.0%)	8 (5.9%)
Not sure	10 (14.1%)	41 (11.8%)	18 (17.3%)	28 (10.6%)	5 (9.5%)	37 (13.0%)	14 (10.4%)
*p*	0.581		0.359			0.093	

Regarding the influence of age on responses to items related to knowledge of ECC, a statistically significant difference was identified only for item 10 “The administration of fluoride is an effective method for preventing cavities”. Specifically, there was a significant variation between age groups (*p* = 0.020) concerning the belief in fluoride usage as an effective preventive measure against caries. Individuals aged 18–30 were more likely to affirm its effectiveness (65.4% responded “Yes”) compared to those aged 31–40 (53.1% responded “Yes”). Conversely, the 31–40 age group exhibited a higher level of uncertainty, with 36.6% responding “Not sure”, compared to 25.9% in the 18–30 age group.

Regarding the influence of the living environment, a significant result was found only for item 2 “The final permanent tooth typically erupts between the ages of 11 and 12 years”. There was a statistically significant difference in responses based on the living environment (*p* = 0.021), indicating that knowledge about tooth eruption timing varies between urban and rural respondents. Urban respondents were more likely to correctly answer “Yes” (59.1%) compared to rural respondents (52.6%). Rural respondents exhibited a higher rate of uncertainty, with 40.7% answering “Not sure” compared to 28.6% of urban respondents.

No other statistically significant differences were found with regard to gender, age, or living environment in relation to knowledge towards ECC.

### 3.3. Gender, Age, Living Environment and Parental Attitude Regarding ECC

Table 3 presents the results from analyzing responses to items 16–22, which investigated parents’ attitudes towards ECC.

**Table 3:** Distribution of respondents by gender, age, and living environment, and their responses to attitude (A) items.

Answer	Gender		Age			Living Environment	
	Male	Female	18–30 y	31–40 y	>40 y	Urban	Rural
**Item 16: I believe that breastfeeding can cause cavities in my child.**							
Strongly disagree	12 (16.9%)	85 (24.4%)	22 (21.2%)	64 (24.4%)	11 (20.8%)	76 (26.8%)	21 (15.6%)
Disagree	22 (31.0%)	113 (32.5%)	33 (31.7%)	83 (31.7%)	19 (35.8%)	91 (32.0%)	44 (32.6%)
Agree	7 (9.8%)	58 (16.7%)	17 (16.3%)	42 (16.0%)	6 (11.3%)	47 (16.6%)	18 (13.3%)
Strongly agree	2 (2.8%)	2 (0.5%)	0 (0.0%)	4 (1.5%)	0 (0.0%)	2 (0.7%)	2 (1.5%)
Not sure	28 (39.5%)	90 (25.9%)	32 (30.8%)	69 (26.4%)	17 (32.1%)	68 (23.9%)	50 (37.0%)
*p*	0.037		0.791			0.019	
**Item 17: I believe that sweets can cause cavities in my child.**							
Strongly disagree	0 (0.0%)	1 (0.3%)	0 (0.0%)	0 (0.0%)	0 (0.0%)	0 (0.0%)	1 (0.7%)
Disagree	1 (1.4%)	6 (1.7%)	0 (0.0%)	0 (0.0%)	0 (0.0%)	5 (1.8%)	2 (1.5%)
Agree	40 (56.3%)	163 (46.8%)	43 (41.3%)	121 (46.2%)	30 (56.6%)	126 (44.4%)	77 (57.0%)
Strongly agree	30 (42.3%)	173 (49.7%)	59 (56.7%)	137 (52.3%)	23 (43.4%)	150 (52.8%)	53 (39.3%)
Not sure	0 (0.0%)	5 (1.5%)	2 (2.0%)	4 (1.5%)	0 (0.0%)	3 (1.0%)	2 (1.5%)
*p*	0.552		0.961			0.067	
**Item 18: I believe that brushing is very important for my child.**							
Strongly disagree	0 (0.0%)	1 (0.3%)	0 (0.0%)	0 (0.0%)	0 (0.0%)	0 (0.0%)	1 (0.7%)
Disagree	0 (0.0%)	2 (0.6%)	0 (0.0%)	0 (0.0%)	0 (0.0%)	1 (0.4%)	1 (0.7%)
Agree	31 (43.7%)	105 (30.2%)	30 (28.8%)	85 (32.4%)	21 (39.6%)	79 (27.8%)	57 (42.2%)
Strongly agree	40 (56.3%)	235 (67.5%)	73 (70.2%)	175 (66.8%)	32 (60.4%)	204 (71.8%)	71 (52.6%)
Not sure	0 (0.0%)	5 (1.4%)	1 (1.0%)	2 (0.8%)	0 (0.0%)	0 (0.0%)	5 (3.8%)
*p*	0.194		0.157			0.000	
**Item 19: I believe that fluoride is a safe method for preventing dental cavities.**							
Strongly disagree	1 (1.4%)	7 (2.0%)	6 (5.8%)	28 (10.7%)	5 (9.4%)	6 (2.1%)	1 (0.7%)
Disagree	5 (7.0%)	30 (8.6%)	0 (0.0%)	0 (0.0%)	0 (0.0%)	26 (9.2%)	10 (7.4%)
Agree	22 (31.0%)	150 (43.1%)	46 (44.2%)	107 (40.8%)	19 (35.8%)	123 (43.3%)	49 (36.3%)
Strongly agree	8 (11.3%)	41 (11.8%)	15 (14.4%)	32 (12.2%)	6 (11.3%)	40 (14.0%)	10 (7.4%)
Not sure	35 (49.3%)	120 (34.5%)	37 (35.6%)	95 (36.3%)	23 (43.5%)	89 (31.4%)	65 (48.2%)
*p*	0.167		0.771			0.012	
**Item 20: I believe that regular dental visits for my child should be adhered to.**							
Strongly disagree	0 (0.0%)	1 (0.3%)	0 (0.0%)	0 (0.0%)	1 (1.9%)	0 (0.0%)	1 (0.7%)
Disagree	1 (1.4%)	3 (0.9%)	0 (0.0%)	0 (0.0%)	0 (0.0%)	1 (0.4%)	1 (0.7%)
Agree	38 (53.5%)	160 (46.0%)	43 (41.3%)	122 (46.6%)	30 (56.6%)	118 (41.5%)	77 (57.0%)
Strongly agree	31 (43.7%)	174 (50.0%)	59 (56.7%)	137 (52.3%)	22 (41.5%)	162 (57.0%)	54 (40.0%)
Not sure	1 (1.4%)	10 (2.8%)	2 (2.0%)	3 (1.1%)	0 (0.0%)	3 (1.1%)	2 (1.6%)
*p*	0.420		0.884			0.054	
**Item 21: I believe that the dentist’s advice regarding my child’s oral health should always be followed.**							
Strongly disagree	0 (0.0%)	2 (0.6%)	0 (0.0%)	0 (0.0%)	0 (0.0%)	1 (0.4%)	1 (0.7%)
Disagree	2 (2.8%)	0 (0.00%)	0 (0.0%)	0 (0.0%)	0 (0.0%)	2 (0.7%)	1 (0.7%)
Agree	39 (54.9%)	148 (42.5%)	38 (36.5%)	109 (41.6%)	24 (45.3%)	115 (40.5%)	61 (45.3%)
Strongly agree	30 (42.3%)	194 (55.7%)	65 (62.5%)	151 (57.6%)	28 (52.8%)	163 (57.4%)	71 (52.6%)
Not sure	0 (0.0%)	4 (1.2%)	1 (1.0%)	2 (0.8%)	1 (1.9%)	3 (1.0%)	1 (0.7%)
*p*	0.005		0.808			0.122	
**Item 22: I believe that when cavities appear, they should be treated immediately, even in primary teeth.**							
Strongly disagree	0 (0.0%)	1 (0.3%)	0 (0.0%)	1 (0.4%)	0 (0.0%)	0 (0.0%)	1 (0.7%)
Disagree	3 (4.2%)	5 (1.4%)	5 (4.8%)	2 (0.8%)	1 (1.9%)	6 (2.1%)	2 (1.5%)
Agree	32 (45.1%)	150 (43.2%)	34 (32.7%)	124 (47.3%)	24 (45.3%)	121 (42.6%)	61 (45.2%)
Strongly agree	33 (46.5%)	172 (49.4%)	56 (53.8%)	124 (47.3%)	25 (47.2%)	138 (48.6%)	67 (49.6%)
Not sure	3 (4.2%)	20 (5.7%)	9 (8.7%)	11 (4.2%)	3 (5.6%)	19 (6.7%)	4 (3.0%)
*p*	0.556		0.080			0.312	

In terms of gender, statistically significant differences were observed for items 16 (*p* = 0.037) and 21 (*p* = 0.005), as outlined below:

- Item 16—“I believe that breastfeeding can cause cavities in my child”: Gender influenced the certainty or agreement with the belief that breastfeeding can cause tooth decay. Female respondents exhibited higher certainty in disagreeing with this statement, with 56.9% either disagreeing or strongly disagreeing, compared to 47.9% of male respondents. Male respondents showed a notably higher level of uncertainty, with 39.5% responding “Not sure”, compared to 25.9% of female respondents.
- Item 21—“I believe that the dentist’s advice regarding my child’s oral health should always be followed”: Gender also affected the certainty or agreement regarding the necessity of always respecting a dentist’s advice on a child’s oral health. Female respondents demonstrated a higher level of certainty in strongly agreeing with this statement, with 55.7% strongly agreeing, compared to 42.3% of male respondents. Male respondents showed a slightly higher level of general agreement (54.9%) compared to female respondents (42.5%), although female respondents exhibited a stronger overall conviction.

As for the living environment, statistically significant results were identified for items 16 (*p* = 0.019), 18 (*p* = 0.000), and 19 (*p* = 0.012), as outlined below:

- Item 16—“I believe that breastfeeding can cause cavities in my child”: The living environment influenced beliefs about the relationship between breastfeeding and cavities. Urban respondents were more likely to disagree or strongly disagree with this statement, with 58.8% combined, compared to 48.2% of rural respondents. Rural respondents showed a higher rate of uncertainty, with 37.0% responding “Not sure,” compared to 23.9% of urban respondents.
- Item 18—“I believe that brushing is very important for my child”: The living environment strongly influenced beliefs about the importance of tooth brushing. Urban respondents were more likely to strongly agree with the importance of tooth brushing, with 71.8%, compared to 52.6% of rural respondents. Although both groups overwhelmingly agreed or strongly agreed with the statement, urban residents displayed a stronger conviction. Rural respondents exhibited slightly more diversity in their responses, including some uncertainty (3.8% answered “Not sure”), which was absent in the urban group.
- Item 19—“I believe that fluoride is a safe method for preventing dental cavities”: The living environment also influenced beliefs regarding the safety of fluoride. Urban respondents were more likely to agree or strongly agree with fluoride safety, with 57.4% combined, compared to 43.7% of rural respondents. Rural respondents showed a higher rate of uncertainty, with 48.2% responding “Not sure”, compared to 31.4% of urban respondents.

No other statistically significant differences were identified beyond those mentioned.

### 3.4. Gender, Age, Living Environment and Parental Practice Regarding ECC

Regarding the influence of gender on responses to items related to parental practices concerning ECC, statistically significant results were identified for items 24 (*p* = 0.035), 25 (*p* = 0.005), 31 (*p* = 0.013), and 32 (*p* = 0.033), as outlined below:

- Item 24—“Brushing is done easily, without any protests from my child”: Gender affected the ease with which parents can brush their child’s teeth without resistance. Female respondents were more likely to report that they often brush their child’s teeth without protest (41.7%) compared to male respondents (59.2%). Conversely, male respondents reported a higher percentage of being able to sometimes brush their child’s teeth without protest (22.5%) compared to female respondents (27.0%).
- Item 25—“I use fluoride toothpaste for my child”: Gender influenced the frequency of using fluoride toothpaste for children. Female respondents were more likely to use fluoride toothpaste frequently (44.0%) compared to male respondents (39.4%). However, male respondents exhibited a higher level of uncertainty regarding fluoride toothpaste use, with 25.4% responding “Not sure” compared to 10.1% of female respondents.
- Item 31—“My child drinks sweetened beverages from a bottle (tea, milk)”: Gender affected the frequency with which children drink sugared beverages from a bottle. Female respondents were more likely to report that their child never consumes sugared beverages from a bottle (68.1%) compared to male respondents (46.5%). Conversely, male respondents reported a higher percentage of sometimes allowing sugared beverages (38.0%) compared to female respondents (20.9%).
- Item 32—“If I notice cavities or other oral issues in my child, I immediately schedule an appointment with the dentist”: Gender influenced the promptness of scheduling a dental appointment for a child with oral issues. Female respondents were more likely to always schedule an appointment immediately (63.5%) compared to male respondents (50.7%). However, male respondents reported a higher percentage of often scheduling appointments (39.4%) compared to female respondents (29.9%).

Statistically significant differences were observed between age and responses to item 23 “I always manage to brush my child’s teeth twice a day” (*p* = 0.040). Specifically, individuals aged 18–30 years are more likely to report brushing their child’s teeth twice a day frequently (65.4%) compared to those aged 31–40 years (53.0%) and those over 40 years (43.4%). However, individuals over 40 years report a higher percentage of brushing twice a day always (26.4%) compared to individuals aged 18–30 years (14.4%) and 31–40 years (13.0%).

Regarding the influence of the living environment on responses in this category, statistically significant differences were found only for item 27 “I regularly check my child’s teeth” (*p* = 0.009). The living environment affects the frequency of checking children’s teeth. While both urban and rural respondents most commonly report checking “Often”, urban respondents are more likely to report checking “Always” (29.6%) compared to rural respondents (16.3%). Conversely, rural respondents show a higher rate of checking “Sometimes” (20.7%) compared to urban respondents (11.6%).

These are the only statistically significant results identified. Table 4 presents the results from this category according to gender, age, and living environment.

**Table 4:** Distribution of respondents by gender, age, and living environment, and their responses to practice (P) items.

Answer	Gender		Age			Living Environment	
	Male	Female	18–30 y	31–40 y	>40 y	Urban	Rural
**Item 23: I always manage to brush my child’s teeth twice a day.**							
Never	1 (1.4%)	7 (2.0%)	3 (2.9%)	4 (1.5%)	1 (1.9%)	6 (2.1%)	2 (1.5%)
Sometimes	15 (21.1%)	78 (22.4%)	15 (14.4%)	67 (25.6%)	11 (20.8%)	66 (23.2%)	27 (20.0%)
Often	40 (56.3%)	190 (54.6%)	68 (65.4%)	139 (53.0%)	23 (43.4%)	143 (50.4%)	87 (64.4%)
Always	11 (15.5%)	52 (14.9%)	15 (14.4%)	34 (13.0%)	14 (26.4%)	49 (17.3%)	14 (10.4%)
Not sure	4 (5.7%)	21 (6.1%)	3 (2.9%)	18 (6.9%)	4 (7.5%)	20 (7.0%)	5 (3.7%)
*p*	0.995		0.040			0.074	
**Item 24: Brushing is done easily, without any protests from my child.**							
Never	0 (0.0%)	11 (3.1%)	2 (1.9%)	8 (3.1%)	1 (1.9%)	9 (3.2%)	2 (1.5%)
Sometimes	16 (22.5%)	94 (27.0%)	26 (25.0%)	71 (27.1%)	13 (24.5%)	75 (26.4%)	35 (25.9%)
Often	42 (59.2%)	145 (41.7%)	45 (43.3%)	121 (46.1%)	21 (39.6%)	121 (42.6%)	66 (48.9%)
Always	9 (12.6%)	83 (23.9%)	25 (24.0%)	51 (19.5%)	16 (30.2%)	65 (22.9%)	27 (20.0%)
Not sure	4 (5.7%)	15 (4.3%)	6 (5.8%)	11 (4.2%)	2 (3.8%)	14 (4.9%)	5 (3.7%)
*p*	0.035		0.836			0.652	
**Item 25: I use fluoride toothpaste for my child.**							
Never	2 (2.8%)	30 (8.6%)	7 (6.7%)	12 (4.6%)	0 (0.0%)	23 (8.1%)	9 (6.7%)
Sometimes	7 (9.9%)	52 (14.9%)	12 (11.5%)	43 (16.5%)	8 (15.1%)	34 (12.0%)	25 (18.5%)
Often	28 (39.4%)	153 (44.0%)	57 (54.8%)	142 (54.2%)	25 (47.2%)	121 (42.6%)	60 (44.4%)
Always	16 (22.5%)	78 (22.4%)	18 (17.4%)	63 (24.1%)	17 (32.0%)	69 (24.3%)	25 (18.5%)
Not sure	18 (25.4%)	35 (10.1%)	10 (9.6%)	12 (4.6%)	3 (5.7%)	37 (13.0%)	16 (11.9%)
*p*	0.005		0.236			0.335	
**Item 26: I take my child to the dentist for regular check-ups.**							
Never	2 (2.8%)	30 (8.6%)	4 (3.8%)	3 (1.1%)	0 (0.0%)	5 (1.8%)	2 (1.5%)
Sometimes	7 (9.9%)	52 (14.9%)	7 (6.8%)	36 (13.7%)	8 (15.1%)	33 (11.6%)	18 (13.3%)
Often	28 (39.4%)	153 (44.0%)	60 (57.7%)	124 (47.3%)	23 (43.4%)	136 (47.9%)	71 (52.7%)
Always	16 (22.5%)	78 (22.4%)	29 (27.9%)	92 (35.1%)	20 (37.7%)	102 (35.9%)	39 (28.9%)
Not sure	18 (25.4%)	35 (10.1%)	4 (3.8%)	7 (2.8%)	2 (3.8%)	8 (2.8%)	5 (3.7%)
*p*	0.411		0.168			0.690	
**Item 27: I regularly check my child’s teeth.**							
Never	0 (0.0%)	7 (2.0%)	2 (1.9%)	2 (0.8%)	0 (0.0%)	2 (0.7%)	2 (1.5%)
Sometimes	11 (15.5%)	40 (11.5%)	10 (9.6%)	43 (16.4%)	8 (15.1%)	33 (11.6%)	28 (20.7%)
Often	39 (54.9%)	166 (47.7%)	63 (60.6%)	142 (54.2%)	25 (47.2%)	151 (53.2%)	79 (58.5%)
Always	19 (26.8%)	124 (35.6%)	26 (25.0%)	63 (24.0%)	17 (32.1%)	84 (29.6%)	22 (16.3%)
Not sure	2 (2.8%)	11 (3.2%)	3 (2.9%)	12 (4.6%)	3 (5.7%)	14 (4.9%)	4 (3.0%)
*p*	0.239		0.526			0.009	
**Item 28: My child eats sweets only during main meals.**							
Never	10 (14.1%)	52 (14.9%)	19 (18.3%)	40 (15.3%)	3 (5.7%)	2 (0.7%)	2 (1.5%)
Sometimes	32 (45.1%)	171 (49.1%)	44 (42.3%)	129 (49.2%)	30 (56.6%)	33 (11.6%)	28 (20.7%)
Often	16 (22.5%)	78 (22.5%)	25 (24.0%)	60 (22.9%)	9 (17.0%)	151 (53.2%)	79 (58.5%)
Always	1 (1.4%)	18 (5.2%)	4 (3.8%)	10 (3.8%)	5 (9.4%)	84 (29.6%)	22 (16.3%)
Not sure	12 (16.9%)	29 (8.3%)	12 (11.4%)	23 (8.8%)	6 (11.3%)	14 (4.9%)	4 (3.0%)
*p*	0.165		0.249			0.542	
**Item 29: My child is still breastfed.**							
Never	21 (29.6%)	149 (42.8%)	35 (33.7%)	110 (42.0%)	25 (47.2%)	2 (0.7%)	2 (1.5%)
Sometimes	19 (26.8%)	53 (15.2%)	19 (18.3%)	44 (16.8%)	9 (17.0%)	33 (11.6%)	28 (20.7%)
Often	19 (26.8%)	81 (23.3%)	29 (27.9%)	63 (24.0%)	8 (15.1%)	151 (53.2%)	79 (58.5%)
Always	11 (15.4%)	56 (16.1%)	17 (16.3%)	39 (14.9%)	11 (20.7%)	84 (29.6%)	22 (16.3%)
Not sure	1 (1.4%)	9 (2.6%)	4 (3.8%)	6 (2.3%)	0 (0.0%)	14 (4.9%)	4 (3.0%)
*p*	0.101		0.473			0.123	
**Item 30: My child drinks unsweetened milk from a bottle.**							
Never	21 (29.6%)	143 (41.1%)	32 (30.8%)	111 (42.4%)	21 (39.6%)	2 (0.7%)	2 (1.5%)
Sometimes	15 (21.1%)	41 (11.8%)	13 (12.5%)	36 (13.7%)	7 (13.2%)	33 (11.6%)	28 (20.7%)
Often	23 (32.4%)	86 (24.7%)	31 (29.8%)	64 (24.4%)	14 (26.4%)	151 (53.2%)	79 (58.5%)
Always	10 (14.1%)	70 (20.1%)	23 (22.1%)	47 (18.0%)	10 (18.9%)	84 (29.6%)	22 (16.3%)
Not sure	2 (2.8%)	8 (2.3%)	5 (4.8%)	4 (1.5%)	1 (1.9%)	14 (4.9%)	4 (3.0%)
*p*	0.079		0.475			0.828	
**Item 31: My child drinks sweetened beverages from a bottle (tea, milk).**							
Never	33 (46.5%)	237 (68.1%)	64 (61.5%)	168 (64.1%)	38 (71.7%)	2 (0.7%)	2 (1.5%)
Sometimes	27 (38.0%)	73 (20.9%)	24 (23.1%)	66 (25.2%)	10 (18.8%)	33 (11.6%)	28 (20.7%)
Often	7 (9.9%)	22 (6.4%)	11 (10.6%)	15 (5.7%)	3 (5.7%)	151 (53.2%)	79 (58.5%)
Always	2 (2.8%)	8 (2.3%)	2 (1.9%)	7 (2.7%)	1 (1.9%)	84 (29.6%)	22 (16.3%)
Not sure	2 (2.8%)	8 (2.3%)	3 (2.9%)	6 (2.3%)	1 (1.9%)	14 (4.9%)	4 (3.0%)
*p*	0.013		0.816			0.741	
**Item 32: If I notice cavities or other oral issues in my child, I immediately schedule an appointment with the dentist.**							
Never	2 (2.8%)	2 (0.6%)	1 (1.0%)	3 (1.1%)	0 (0.0%)	2 (0.7%)	2 (1.5%)
Sometimes	1 (1.4%)	14 (4.0%)	4 (3.8%)	9 (3.4%)	2 (3.8%)	33 (11.6%)	28 (20.7%)
Often	28 (39.4%)	104 (29.9%)	33 (31.7%)	83 (31.7%)	16 (30.2%)	151 (53.2%)	79 (58.5%)
Always	36 (50.7%)	221 (63.5%)	63 (60.6%)	161 (61.5%)	33 (62.2%)	84 (29.6%)	22 (16.3%)
Not sure	4 (5.7%)	7 (2.0%)	3 (2.9%)	6 (2.3%)	2 (3.8%)	14 (4.9%)	4 (3.0%)
*p*	0.033		0.998			0.387	

## 4. Discussion

Given the increasing global prevalence of dental caries among the pediatric population [8], alongside the World Health Organization’s growing efforts to eliminate dental caries in children [21], and recognizing the importance of establishing healthy oral and dental habits within the family context [22], this study aimed to provide valuable insights into gender, age, and environmental differences in parental KAP concerning ECC in Bihor, Romania. The focus on this particular region is warranted, as no previous studies have addressed the issue of ECC in this part of the country. Nationally, research on ECC is limited, and studies that specifically explore parental KAP related to ECC are absent. The findings from this study reveal significant disparities, underscoring the need for targeted educational interventions to bridge these gaps.

A KAP-type questionnaire was utilized in this study, as it enables the investigation of several essential components within a single instrument, providing a comprehensive overview of the selected topic [14]. The questionnaire was adapted from the work of Mani et al. (2012) [19], translated into Romanian, and used without creating a new questionnaire. This approach was justified by the clarity and comprehensiveness of the existing questions, which effectively captured various aspects relevant to ECC.

Although additional variables were investigated, this study focused specifically on gender, age, and environment of origin. This decision was made due to the large volume of data and the necessity of thoroughly analyzing each variable to identify statistically significant differences. As a result, this paper represents the first part of a broader study on parental KAP regarding ECC in Bihor, Romania. These three variables were prioritized because they are generally considered the most important and frequently examined factors across studies, regardless of the research topic [23].

Gender differences were evident in the responses, with female participants demonstrating greater certainty regarding several key aspects, such as awareness of the pain children experience from dental caries, the importance of brushing twice daily, and the lack of a relationship between breastfeeding and tooth decay. These findings are consistent with previous research, which has similarly reported gender-based differences in parental knowledge and practices [16,24]. Al-Jaber et al. (2002) administered a similar questionnaire to respondents in Qatar and found that female respondents demonstrated higher percentages of correct responses compared to their male counterparts [15]. Consistent with these findings, the present study revealed that female participants exhibited higher levels of knowledge, with a statistically significant difference favoring female participants over males in several areas. This pattern, where mothers appear to be more informed than fathers and display better attitudes and practices toward ECC prevention, is also corroborated by Nassar et al. (2022) [16]. Similar to the findings of the current study, Nassar et al. (2022) emphasize that female respondents are more knowledgeable, possess more favorable attitudes towards ECC prevention, and exhibit superior oral hygiene practices compared to male respondents [16]. However, other studies have found no statistically significant influence of parental gender on responses, even though mothers tended to be more informed [25]. Male respondents, on the other hand, exhibited higher levels of uncertainty, highlighting the need for more targeted educational efforts directed towards fathers. This disparity may be explained by the fact that mothers typically assume the primary caregiving role and are more likely to engage in health-related discussions, research, and decision-making concerning their children’s health [26,27]. Additionally, gender differences were observed in parental practices, with mothers being more likely to brush their child’s teeth without resistance, use fluoride toothpaste, and promptly schedule dental appointments for oral health issues. These findings emphasize the importance of incorporating gender-specific strategies in public health initiatives aimed at improving oral health outcomes [11,28].

Age differences were also evident, particularly regarding beliefs about the effectiveness of fluoride in preventing dental caries. Younger parents (aged 18–30) were more likely to affirm fluoride’s effectiveness, a finding consistent with previous studies that have highlighted the influence of age on parental knowledge and attitudes [29]. One possible explanation for younger parents’ better access to information may be their familiarity with the digital age. Younger parents often have greater access to up-to-date health information through the internet, health apps, and social media, allowing them to quickly learn and implement best practices for their children’s health, including dental care [30]. In contrast, older parents may rely more heavily on traditional sources of advice, which can sometimes be outdated, and may experience higher levels of stress, which could impede their ability to stay informed about current health recommendations [31].

The living environment significantly influenced parental knowledge and attitudes. Urban parents were more likely to correctly answer questions regarding the timing of tooth eruption and exhibited stronger convictions about the safety of fluoride and the importance of tooth brushing. This aligns with studies that have documented similar urban-rural disparities in parental KAP [32,33]. In rural areas, there is often limited emphasis on improving oral health, compounded by challenges such as fewer dental offices and higher treatment costs. Consequently, preventive care is less prevalent in these communities, leading to a greater reliance on curative treatments [34,35].

These findings have important implications for public health policy and practice. Developing targeted educational interventions that address demographic factors such as gender, age, and living environment could help mitigate the incidence of ECC and enhance oral health outcomes in the Bihor region of Romania. Beyond the immediate negative effects of early childhood caries (ECC), the condition can have medium- and long-term consequences, extending into adolescence and adulthood. The early extraction of primary molars due to complications from dental caries can result in the misalignment of premolars [36], while the destruction or early extraction of primary incisors may contribute to the development of harmful oral habits that exacerbate dento-maxillary anomalies [37]. Furthermore, the first permanent molar, which plays a critical role in establishing proper occlusion, is particularly susceptible to caries if it erupts in an oral environment already compromised by ECC [38]. This tooth frequently develops severe caries, often requiring endodontic intervention during adolescence [39] and prosthetic treatments in adulthood [40]. Additionally, the correction of malocclusions necessitates orthodontic treatment, followed by prolonged retention to maintain the results [41,42]. These considerations underscore the significant burden that ECC imposes over time [43]. Therefore, a thorough understanding of parental KAP is essential for creating effective strategies to promote preventive oral health behaviors and reduce the long-term healthcare burden associated with untreated dental caries [43].

The study has several limitations that should be considered when interpreting the results. First, the use of an online KAP questionnaire may have introduced selection bias, as only parents with access to the internet and social media platforms were able to participate. This may exclude individuals from lower socioeconomic backgrounds or those in rural areas with limited internet access, potentially affecting the generalizability of the findings. Additionally, the reliance on self-reported data could lead to response bias, where participants may have overestimated or underestimated their knowledge, attitudes, or practices related to early childhood caries. Lastly, while the study aimed to address gaps in the literature specific to Romania, the regional focus on Bihor may not fully represent the diversity of parental experiences across the country. Future studies should aim to include a more geographically diverse sample to better assess changes over time in parental knowledge and practices regarding childhood oral health.

Finally, it is important to note the fact that based on the findings presented, the hypothesis that demographic factors such as gender, age, and living environment influence, at least in some measure, the KAP of parents in Bihor, Romania, regarding ECC is supported by the data. The results show multiple statistically significant differences between demographic groups in their responses to various items assessing knowledge, attitudes, and practices related to ECC, which leads to the acceptance of the hypothesis.

## 5. Conclusions

In conclusion, this study highlights some significant disparities in parental KAP regarding ECC in Bihor, Romania, based on gender, age, and living environment. Female participants generally demonstrated higher knowledge and stronger oral health practices compared to males, while younger parents were more informed about the benefits of fluoride. Urban parents showed a greater understanding of oral hygiene and preventive care than their rural counterparts. These findings emphasize the need for targeted educational interventions that address these demographic differences to improve oral health outcomes in young children. Public health initiatives should consider gender-specific approaches and focus on enhancing access to preventive dental care, particularly in rural areas. By improving parental knowledge and practices, especially among fathers and rural communities, the long-term burden of ECC can be reduced, contributing to better oral health for future generations.

## Data Availability

The data presented in this study are available upon request from the corresponding authors due to ethical reasons.
